# Longer Drug Retention of Interleukin-12/23 or Interleukin-17 Inhibitors Compared With TNF Inhibitors in Female Patients With TNF Inhibitor-Experienced Psoriatic Arthritis

**DOI:** 10.1016/j.mayocpiqo.2025.100622

**Published:** 2025-05-12

**Authors:** Godehard A. Scholz, Eleftherios Papagiannoulis, Christoph Blapp, Raphael Micheroli, Adrian Ciurea, Michael J. Nissen, Nikhil Yawalkar, Diana Dan, Jennifer Amsler, Almut Scherer, Burkhard Möller

**Affiliations:** a Department of Rheumatology and Immunology, Inselspital, Bern University Hospital, University of Bern, Switzerland; b Department of Dermatology, Inselspital, Bern University Hospital, University of Bern, Switzerland; c Swiss Clinical Quality Management in Rheumatic Diseases (SCQM) Foundation, Zurich, Switzerland; d Department of Rheumatology, University Hospital Zurich, Switzerland; e Department of Rheumatology, University Hospital of Geneva, Switzerland; f Department of Rheumatology, Lausanne University Hospital (CHUV) and University of Lausanne, Switzerland

## Abstract

**Objective:**

To compare the effectiveness of Interleukin (IL)-12/23 or IL-17A inhibitors (summarized to inhibitors of the Th17 cell generation or function, Th17i) with tumor necrosis factor inhibitors (TNFi) in patients with TNFi-experienced psoriatic arthritis (PsA).

**Patients and Methods:**

We conducted a comparative effectiveness study by taking advantage of prospectively collected patients with PsA data from the Swiss Clinical Quality Management in Rheumatic Diseases register, encompassing the interval from January 1, 2015 to August 1, 2021. Drug retention was the primary outcome in unadjusted and inverse propensity-weighted Cox regression models. Secondary outcomes were a static skin score for psoriasis, the American College of Rheumatology (ACR) 20, 50, and 70 response rates, and the disease activity in PsA score.

**Results:**

At baseline, Th17i (n=341) were initiated in patients with more severe skin disease, but with comparable disease activity as TNFi (n=503) in all other disease domains. In the unadjusted analysis, Th17i were later discontinued than TNFi (median 828 vs 445 days, *P*<.001), but the hazard ratio for discontinuation was significantly lower for Th17i than for TNFi only in women (0.57 [0.37-0.87], *P*=.01). Furthermore, differences in static skin scores between the groups at baseline were equalized at follow-up. However, improvements in the disease activity in PsA were similar in both groups, and ACR20 (33% [29%] vs 14% [13%]; *P*=.03) and ACR50 response rates (24% [21%] vs 7% [6%]; *P*=.02) were even higher for TNFi in unadjusted and (LUNDEX-adjusted) analyses.

**Conclusion:**

After TNFi failure, more profound skin improvement and longer drug retention in women argue in favor of switching to Th17i in certain patient populations. However, TNFi may at least be equivalent in improving locomotor system manifestations and remain a viable option in the first and in the later treatment line of PsA.

Psoriatic arthritis (PsA) is an immune-mediated inflammatory condition, which may affect the skin, nails, peripheral joints, spine, tendons, and entheses.^1^ Tumor necrosis factor (TNF) emerged as the first pivotal cytokine implicated at all these sites in patients with PsA, establishing TNF inhibitors (TNFi) as the first class of target-specific biological Disease-Modifying Anti-Rheumatic Drugs (bDMARDs) for all PsA manifestations. The TNFi are the only target-defined bDMARD class backed by proven efficacy in head-to-head comparisons with conventional synthetic (cs) DMARDs,^2^ which, together with lower costs post-patent expiry of several TNFis, highlight their current role as a prominent choice among the bDMARDs.^3^, ^4^, ^5^

The identification of interleukin (IL)-17 and its subtypes historically succeeded the identification of TNF as another pivotal proinflammatory cytokine in PsA. Subsequently, the IL-23/17 axis was defined by T cells directed to Th17 memory cells upon IL-23 receptor stimulation, which became another key target for further bDMARD development.^6^ First, anti-IL-17A and p40-specific anti-IL-23 bDMARDs were developed, the latter being a component present in 2 proinflammatory mediators, IL-23 and IL-12. In the following, IL-17A and p40-specific antibodies were the first representatives of Th17i that were shown to be superior to TNFi for the treatment of skin psoriasis,^7^^,^^8^ an effect amplified with newer representatives of inhibitors of the IL-23/17 axis.^9^^,^^10^ Although TNFi may be equally potent in efficiently treating various disease domains of PsA, priority for inhibition of IL-23/17 is currently limited to severe skin disease.^3^, ^4^, ^5^ However, the broader treatment goal for PsA remains achieving the lowest possible disease activity across all domains. It is increasingly acknowledged that TNFi achieve lower treatment continuation rates in women than in men,^11^ while sex-related differences in the effectiveness of Th17i are currently not known. Real-world data for TNFi and Th17i in different PsA treatment lines is expanding;^12^, ^13^, ^14^, ^15^, ^16^, ^17^ however, definitive recommendations for their position in later bDMARD treatment stages remain to be defined.^3^, ^4^, ^5^

Given the absence of reliable predictors for treatment response of bDMARDs for different targets, this study aims to identify clinical factors optimizing treatment effectiveness, particularly in the common scenario of choosing between another TNFi and Th17i for TNFi-experienced patients with PsA.

## Methods

### Study Design

A comparative effectiveness study was conducted using prospectively collected data from patients with PsA in the Swiss Clinical Quality Management in Rheumatic Diseases (SCQM) register.^18^ Participation in SCQM is restricted to board-certified rheumatologists and their patients and is on a voluntary basis. Although many data were contributed by the rheumatology departments of 5 academic centers and other prominent Swiss hospitals, another large proportion of data originated from private rheumatology practices. The SCQM guidelines advocate for at least annual follow-up visits, with additional interim visits encouraged on initiating a new bDMARD and 3 months post treatment initiation.

### Study Population

Treatment courses (TC) involving the specified bDMARDs were considered for potential inclusion if at least 1 cycle of TNFi had previously been discontinued for any reason. Another inclusion criterion was the initiation of treatment after the introduction of the first Th17i in Switzerland, established on January 1, 2015. The dataset, collected on August 1, 2021, adheres to the Strengthening the Reporting of Observational Studies in Epidemiology (STROBE) guidelines,^19^ the recently published considerations for comparative effectiveness studies by the European Alliance of Associations for Rheumatology,^20^ and the ethical principles outlined in the Declaration of Helsinki. Approval for this study was granted by the cantonal ethics committee in Bern (KEK ID 2019-00968), and all participants provided written informed consent.

### Exposure Variables and Covariates

The studied TNFis were adalimumab, certolizumab pegol, etanercept, golimumab, and infliximab including their biosimilars. Patients in the Th17i treatment group received the anti-p40 antibody ustekinumab, a shared component of IL-12 and IL-23, secukinumab or ixekizumab, both targeting IL-17A. As long as a TC fulfilled these inclusion criteria, patients could contribute more than 1 TC to the analysis.

### Outcome Parameters

The primary outcome was the number of days from the start of a bDMARD to its stop date, as recorded by the health care provider, or the start date of another bDMARD or targeted synthetic (ts) DMARD, whichever came first. Observations were censored at the end date of the study or at drug discontinuation due to remission. Treatment interruptions of less than 3 months were ignored and switching from the originator to a biosimilar of the same generic name merged and considered as one ongoing TC.

Secondary outcomes were the proportion of patients meeting the ACR20/50/70 response criteria for the treatment of interest,^21^ analyzed as-observed and in a LUNDEX-adjusted approach.^22^ In the completer population, additional categorical secondary outcomes were calculated on the proportion of patients achieving minimal disease activity (MDA) according to ≥5/7 criteria,^23^ very low disease activity according to 7/7 fulfilled criteria,^24^ remission (≤4 points) and low disease activity (≤14 points) in the disease activity of PsA (DAPSA) score,^25^ good, moderate, or poor improvement categories in DAPSA,^25^ in the disease activity score based on 28 joints and C-reactive protein concentrations (DAS28-CRP),^26^ and the PsA response criteria.^27^ In addition, we analyzed the absolute values in static physician global assessment of skin severity,^28^ Maastricht ankylosing spondylitis enthesitis score (MASES),^29^ DAS28-CRP,^26^ DAPSA, and clinical DAPSA (cDAPSA),^25^ the health assessment questionnaire of disability index,^30^ the questionnaire on health-related quality of life as assessed by the EuroQoL group questionnaire in 5 dimensions (EQ-5D)^31^ and the dermatology life quality index at follow-up.^32^

### Statistical Analyses

Differences in contingency tables were assessed for statistical significance using Fisher’s exact test. The Kruskal-Wallis test was applied to quantitative data. The primary outcome was drug retention. Crude drug retention analyses used the log-rank test. To address baseline covariate imbalances between bDMARD classes at start of treatment, inverse propensity score-weighted (IPW) models were implemented. Propensity scores (PS) were generated by logistic regression based on baseline data for swollen and tender joint counts, modified MASES for PsA,^29^ the static 7-point Likert skin severity scale,^33^^,^^34^ previous bDMARD class and line, and a patient history of dactylitis, inflammatory bowel disease, and uveitis at any time before treatment start. Independence of the main effects of bDMARD class and sex was tested through the generation of a drug type/sex interaction variable. Secondary outcome disease activity parameters were assessed for changes between baseline and the first visit and their absolute values at follow-up visits after 3 to 12 months. In case of more than one available follow-up visit the first one was chosen. The information on important covariates such as body mass index, disease duration, or extra-musculoskeletal manifestations was missing in up to 10% of the therapy cycles, and information on the number of swollen or tender joints, MASES, or severity of skin involvement was missing in up to 25% of cases. We assessed missingness at random based on its distribution across the 2 therapy groups before we calculated estimates by using multiple imputation in chained equations. Because all models with this dataset of 261 TNFi and 184 Th17i did not relevantly differ from as-observed data, we only present the results of the dataset without imputation. The McNemar test was employed to test the significance of differences in disease activity categories at baseline and follow-up. The Kruskal-Wallis test was applied to quantitative data. P-values obtained were not adjusted for multiplicity of testing. Analyses were performed using the statistical package R version 4.3.2.

### Patient and Public Involvement

Since 2023, patients are represented on the steering board of the SCQM. This study was planned and conducted earlier, so patients were not yet actively involved in this study.

## Results

### Treatment Groups

**Figure 1:**
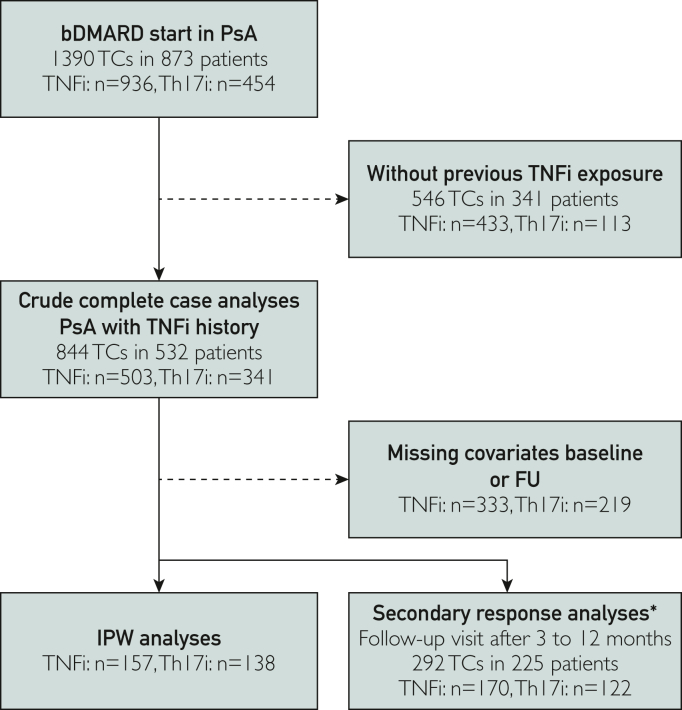
Flow diagram of inclusion for the different primary crude, IPW and secondary outcome analyses, dependent on data availability and treatment allocation. ∗Complete data at baseline and in a follow-up visit 3-12 months later after MICE; numbers may be less, dependent on the specific outcome. bDMARD, biological Disease-Modifying Anti-Rheumatic drug; FU, follow-up; Th17i, inhibitors of the Th17 cell generation or function; IPW, inverse propensity score-weighted; MICE, multiple imputation in chained equations; PsA, psoriatic arthritis; TCs, treatment courses; TNFi, tumor necrosis factor inhibitors.

As of the extraction date on August 1, 2021, 1112 patients with a clinical diagnosis of PsA had since January 1, 2015 initiated 1821 bDMARD treatments, consisting of 1276 TNFi TCs and 545 Th17i TCs. For this study, only TCs after one or more previous TNFi exposures were included. Overall, 503 TNFi and 341 Th17i TCs were included into analyses related to the primary outcome (Figure 1).

### Baseline Characteristics

Table 1 provides a comprehensive overview of baseline characteristics. In line with current guidelines, skin disease activity was notably higher at baseline of Th17i when compared with TNFi treatments. The Th17i were more commonly administered to patients with nail involvement. Although MASES was higher at baseline in patients with Th17i, baseline swollen and tender joint counts, and a history of dactylitis, spinal involvement, inflammatory bowel disease, or uveitis, did not show significant differences between Th17i and TNFi TCs. Moreover, Th17i were more frequently used in patients with concomitant cardiovascular disease than TNFi. Treatment history also differed significantly between Th17i and TNFi TCs (Supplemental Table 1, available online at http://www.mcpiqojournal.org). For instance, Th17i were initiated more often after failure of the previous bDMARD due to ineffectiveness rather than side effects, compared with TNFi. The Th17i were less frequently used with concurrent conventional synthetic disease-modifying anti-rheumatic drug co-medication, more commonly in patients with targeted synthetic disease-modifying anti-rheumatic drug experience and in later bDMARD treatment lines.

**Table 1:** Patient Characteristics at Start of 503 TNFi and 341 Th17i TCs^a^^,^^b^

Characteristic	n		TNFi	Th17i	*P*
Age (y)	844	Median (IQR)	51 (41-61)	52 (46-59)	.14
Disease duration (y)	825	Median (IQR)	11 (5-18)	12 (7-19)	.10
Male	844	n (%)	189 (37.6)	134 (39.3)	.61
Female		n (%)	314 (62.4)	207 (60.7)	
BMI (kg/m^2^)	268	Mean ± SD	28.3 ± 5.5	28.3 ± 6.2	.95
HLA-B27 positive	482	n (%)	66 (13.1)	34 (10.0)	.27
CASPAR positive	445	n (%)	170 (33.8)	146 (42.8)	.004
Dactylitis ever	844	n (%)	122 (24.2)	98 (28.7)	.15
Nail manifestations ever	844	n (%)	53 (10.5)	60 (17.6)	.004
Enthesitis ever	844	n (%)	201 (40.0)	155 (45.5)	.12
History of IBD	810	n (%)	15 (3.0)	13 (3.8)	.14
Active skin disease	475	n (%)	164 (32.6)	143 (41.9)	.008
Physician global skin (0-6)^28^	421	Mean ± SD	2.55 ± 1.50	3.27 ± 1.72	.0001
Active dactylitis	844	n (%)	26 (5.2)	22 (6.5)	.45
Active enthesitis	361	n (%)	85 (16.9)	95 (27.9)	.001
Fibromyalgia	844	n (%)	5 (1.0)	9 (2.6)	.1
Uveitis in last 12 mo	844	n (%)	1 (0.2)	0 (0.0)	1
Inflammatory backpain ever	296	n (%)	78 (46)	57 (45)	.81
Cardiovascular event ever	844	n (%)	79 (15.7)	75 (22.0)	.02
Smoking status	212	n (%)	139 (100)	73 (100)	.82
Former smoker		n (%)	56 (11.1)	27 (7.9)	
Current smoker		n (%)	31 (6.2)	19 (5.6)	
Physical activity groups	209	n (%)	137 (100)	72 (100)	.32
No physical activity		n (%)	44 (32)	26 (36)	
<1 h/wk		n (%)	28 (20)	16 (22)	
1 to 2 h/wk		n (%)	43 (8.6)	21 (6.2)	
>2 h/wk		n (%)	22 (4.4)	9 (2.6)	
Positive MDA status	394	n (%)	32 (6.4)	12 (3.0)	.19
MASES	361	Median (IQR)	0 (0-2)	1 (0-3)	.04
Painful out of 66 joints	369	Median (IQR)	3 (1-8)	4 (1-10)	.17
Swollen out of 68 joints	368	Median (IQR)	1 (0-4)	1 (0-4)	.52
HAQ-DI	296	Mean ± SD	0.76 ± 0.62	0.86 ± 0.64	.22
DLQI	292	Median (IQR)	1.5 (0-7.0)	2 (0-8)	.15
DAS28-CRP	364	Median (IQR)	2.75 (1.90-3.70)	3.10 (2.30-3.80)	.04
DAPSA	292	Median (IQR)	17.1 (9.02-29.5)	17.1 (12.5-25.9)	.82
cDAPSA	292	Median (IQR)	17.5 (10.0-30.0)	17.0 (12.0-23.0)	.64
EQ-5D	297	mean ± SD	64.57 ± 21.01	62.33 ± 22.92	.39
SF-12 MCS	202	Mean ± SD	44.63 ± 11.25	41.64 ± 11.23	.07
SF-12 PCS	202	Mean ± SD	39.63 ± 11.02	37.84 ± 9.52	.25

a Abbreviations: BMI, body mass index; CASPAR, classification criteria for psoriatic arthritis; (c)DAPSA, (clinical) disease activity of psoriatic arthritis^25^; DAS28-CRP, disease activity score based on 28 joints and C-reactive protein^26^; DLQI, dermatology life quality index^32^; EQ-5D, European quality of life questionnaire in five dimensions^31^; HAQ-DI, health assessment questionnaire of disability index^30^; HLA, human leukocyte antigen, IBD, inflammatory bowel disease; IQR, interquartile range; MASES, Maastricht ankylosing spondylitis enthesitis score (with inclusion of the proximal plantar fascia insertions)^29^; MDA, minimal disease activity^23^; MCS, mental component score; PCS, physical component score; SF-12, short form questionnaire with 12 items^34^; TCs, treatment courses; TNFi, tumor necrosis factor inhibitors; Th17i, inhibitors of the Th17 cell generation or function.

b Level of significance in differences between groups was calculated by Fisher’s exact or Kruskal-Wallis test. The n indicates the number of available observations for the respective item. Percentages (in parentheses) are the quotient of numbers of patients with the respective trait divided by the number of cases with available information.

### Primary Outcome Analyses

**Figure 2:**
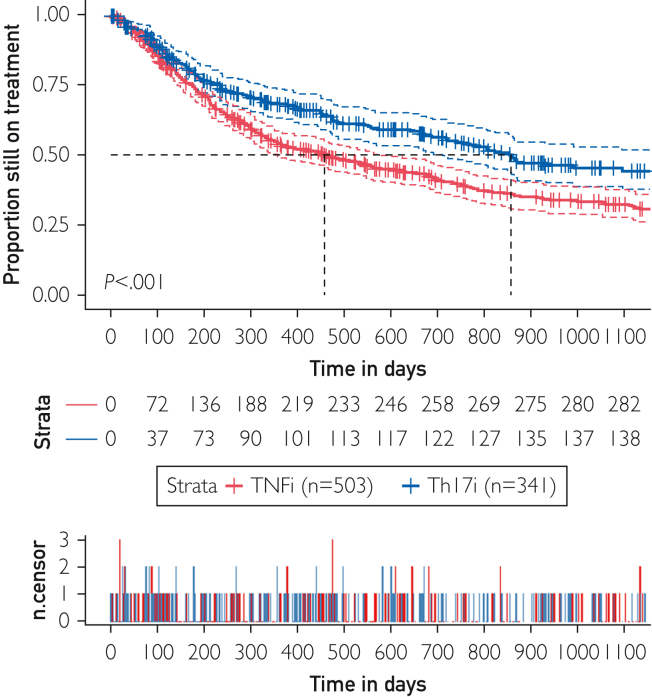
Kaplan-Meyer plots of drug retention in TNFi and Th17i TCs over time, illustrated as the mean (bold lines) and 95% CI (dashed lines). TNFi, tumor necrosis factor inhibitors; Th17i, inhibitors of the Th17 cell generation or function; TCs, treatment courses.

In an unadjusted analysis of the entire cohort (Figure 2), the median time to discontinuation at 860 days was significantly longer for Th17i when compared with 430 days for TNFi (*P*<.001). Competing risk analyses revealed similar patterns of cessation over time for both bDMARD groups (Supplemental Figure 1, available online at http://www.mcpiqojournal.org). In the initial 100 days, discontinuations were similarly frequent due to adverse events for bDMARDs in both categories. At later time, most discontinuations were attributed to ineffectiveness. Discontinuation due to remission occurred only in 4% (n=20) of TNFi TCs and in <1% (n=3) of Th17i TCs. Treatment stop in remission was censored and thus excluded from the discontinuation risk analyses. Furthermore, Kaplan-Meyer curves in women indicated significantly (*P*<.001) earlier discontinuation of TNFi (n=314) than of Th17i (n=207). The same comparison in men (TNFi n=189 and Th17i n=134), in contrast, did not report significantly different drug discontinuation rates (*P*=.14).

**Figure 3:**
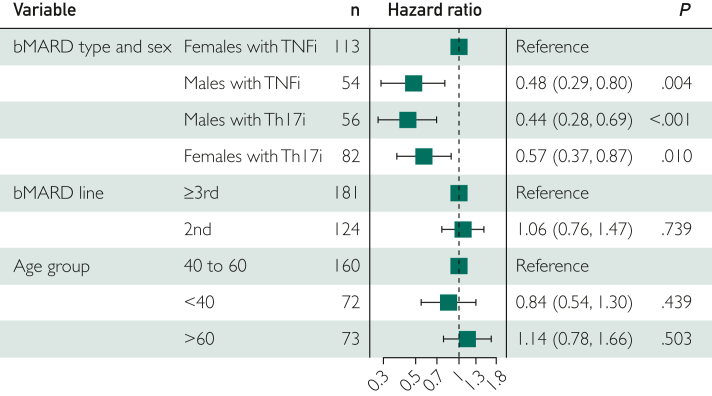
IPW drug retention time model, including an interaction variable between bDMARD type and sex, displaying the proportional hazards of drug discontinuation with their 95% CI for each variable in the model. The PS were derived from the number of swollen and tender joints, MASES^29^, the presence of IBD and uveitis at baseline, current bDMARD line, and class of the previous bDMARD therapy. bDMARD, biological Disease-Modifying Anti-Rheumatic Drug; IBD, inflammatory bowel disease; IPW, inverse propensity score-weighted; MASES, Maastricht ankylosing spondylitis enthesitis score; PS, propensity score; TNFi, tumor necrosis factor inhibitors; Th17i, inhibitors of the Th17 cell generation or function.

To correct for imbalances in the baseline characteristics, we generated PS. This population was well balanced for all weighted variables, but one imbalance persisted for psoriatic nail manifestations and, consequently, classification criteria for PsA positivity (Supplemental Table 2, available online at http://www.mcpiqojournal.org). In an IPW model without interaction, the hazard ratio (HR) for discontinuation of Th17i (n=61 events in 138 exposures) was significantly lower (HR=0.66 [0.47-0.93]; *P*=.02) than for TNFi (reference, 105 events in 157 exposures). Furthermore, women conferred a significantly higher risk of bDMARD discontinuation (HR=1.67 [1.16-2.39]; *P*<.01) than men (reference). The other covariates, including DAS28-CRP, MASES, or static skin activity score, did not alter the comparison of interest. To study effect modification of sex in the comparison between TNFi and Th17i, an interaction variable between these 2 important predictors of earlier discontinuation was included in the IPW model. Of importance, it could be reported in this model that Th17i had a lower HR for discontinuation only in women (HR=0.57 [0.37-0.87]; *P*=.01) (Figure 3).

### Secondary Outcomes Analyses

The ACR20 response rates were low in both groups but significantly higher in TNFi than in Th17i TCs. This finding was true in as-observed (33% (n=22) vs 14% (n=6), *P*=.03) and in LUNDEX-adjusted (29% vs 13%) analyses (Table 2). Furthermore, TNFi also provided significantly better ACR50 response rates than Th17i in as-observed (24% (n=16) vs 7% (n=3); *P*=.02) and in LUNDEX-adjusted treatment comparisons (23% vs 6%). In contrast, ACR70, DAPSA, and cDAPSA low disease activity and remission rates; DAPSA and DAS28-CRP response categories; PsA response criteria; MDA; and very low disease activity rates were not significantly different between TNFi and Th17i (Table 2).

**Table 2:** Secondary Clinical Response Criteria Were Assessed 3 to 12 months After Start of the bDMARD TC of Interest^a^^,^^b^

Clinical response criterium	TNFi		Th17i		*P*
	n	%	n	%	
All cases	170	100	122	100	
ACR response rate	66	100	45	100	
ACR20	22	33	6	14	.03
ACR20 (LUNDEX)		29		13	
ACR50	16	24	3	7	.02
ACR50 (LUNDEX)		21		6	
ACR70	4	6	2	4	1
ACR70 (LUNDEX)		5		4	
DAPSA response	56	100	32	100	.11
Good	4	7	0	0	
Moderate	11	20	3	9	
Poor	41	73	29	91	
DAPSA at follow-up	97	100	49	100	
LDA (<15)	50	52	34	69	1
Remission (<4)	18	19	8	16	.12
DAS28-CRP response	119	100	86	100	.40
Good	19	16	8	9	
Moderate	26	22	21	25	
Poor	74	62	57	66.3	
PsARC response	62	100	40	100	
Yes	27	43	12	30	.21
Minimal disease activity	116	100	85	100	
MDA (≥5/7 criteria)	37	31	21	25	.58
VLDA (7/7 criteria)	9	11	4	5	.57

a Abbreviations: ACR20/50/70, American College of Rheumatology response categories^21^; bDMARD, biological Disease-Modifying Anti-Rheumatic Drug; DAPSA, disease activity in psoriatic arthritis^25^; DAS28-CRP, disease activity score based on 28 joints and C-reactive protein^26^; LDA, low disease activity, based on DAPSA^25^; MDA/VLDA, minimal/very low disease activity^23 24^; n, number of all observations with available data for the specific outcome; PsARC, psoriatic arthritis response criteria^27^; Th17i, inhibitors of the Th17 cell generation or function; TNFi, tumor necrosis factor inhibitors.

b For each variable reported, the number of cases satisfying the respective criterion and percentage refers to the subset of observations with non-missing information relative to the specific criterion. The total number of non-missing observations for the respective outcome in both groups is reported in the first line of each response item.

We observed numerical improvements from baseline to follow-up in CRP, DAPSA, DAS28-CRP, MASES, health assessment questionnaire of disability index, dermatology life quality index, and EQ-5D on treatment with both Th17i and TNFi. However, we only observed a statistically significant improvement in cDAPSA (*P*=.05) in favor of TNFi (mean difference −7.5 (95% CI, −5.96 to −9.04) (Supplemental Table 3, available online at http://www.mcpiqojournal.org). Finally, the absolute measures of disease activity, body function, and health-related quality of life at follow-up after 3 to 12 months were, with a single exception for EQ-5D, numerically more favorable in the TNFi group (Supplemental Table 4, available online at http://www.mcpiqojournal.org).

## Discussion

This study highlights the clinically significant effectiveness of both alternative TNFi and Th17i in patients with PsA with previous TNFi experience in their second or subsequent bDMARD treatment. The Th17i exhibited significantly improved long-term treatment adherence, but only in women. Furthermore, through treatment with Th17i, the initially higher severity of the skin condition was reduced to the same low activity level as with TNFi. However, other secondary outcome analyses did not support any superiority of switching to Th17i than cycling among TNFi. In contrast, when we relinquished to adjust for attrition by IPW but used only LUNDEX-adjustments, completers on TNFi in this study until the first follow-up visit after 3 to 12 months had significantly better ACR20 and ACR50 response rates than had completers on Th17i.

Observational studies come with inherent limitations, notably susceptibility to bias and confounding factors. Although aligning with current PsA management guidelines,^3^, ^4^, ^5^ an example of such a bias is the preferential use of Th17i in patients with more severe skin disease. To address baseline imbalances, we used weighted models with PS, which were generated by 7 disease characteristics plus previous bDMARD class and line. However, we did not include time between from previous TNFi exposure and the treatment of interest, which might also have affected the result. In this study, after introducing an interaction variable, we found evidence for a differential drug retention of Th17i versus TNFi in women. In contrast, we did not see a difference in drug retention between these substance classes in men. This finding appears to a certain extent in line with the current literature, which reports sex-associated disparities in treatment responses to TNFi when used in the first bDMARD line in PsA, other spondyloarthritides and in skin psoriasis.^35^, ^36^, ^37^, ^38^ However, to date, this has not yet been found in reference to bDMARDs with another target. Of note, this finding was distinct from the expected impact on skin severity or other clinical baseline disease characteristics.

Missing data is another constraint of observational studies, which is above all in this study true for the secondary outcome analyses. The extended treatment adherence observed with Th17i in women seems at first glance to be inconsistent with the numerically higher ACR20 and ACR50 response rates seen with TNFi in the initial year of therapy. At a first glance, this finding of the secondary outcome appears contradictory to the primary outcome of treatment adherence. However, ACR response rates were only calculated in completers and only LUNDEX-adjusted. Nevertheless, it is essential to note that the ACR response rates in this real-life study were low and likely less impactful on patient satisfaction than considerably higher figures for the same therapies when applied in clinical PsA trials.^39^, ^40^, ^41^, ^42^ In addition, drug discontinuation occurred in this study in the vast majority at later time points than the assessments for ACR response rates after 3 to 12 months from treatment start. In this study, we were able to examine considerably higher patient numbers regarding the primary outcome compared with the secondary outcome, so we place greater importance on drug retention.

The strategic use of bDMARDs in reflection of a molecular taxonomy has already started,^43^ but the need for therapy guidance in the clinical setting is more urgent than biology-based treatment predictors are to be expected. On the basis of experimental data and for statistical needs in our limited data set,^6^ we grouped one IL-12/23 and 2 IL-17A inhibitors together. This simplification can be justified based on the comparisons of IL-17i and IL-23i subsequent to TNFi in patients with PsA, where no evidence was found for a difference in drug retention between IL-17i and IL-23i.^17^ However, significant differences in the impact of IL-23i and IL-17i on other forms of immune-mediated inflammation, such as in the spine and the intestine may complicate categorizing Th17i as a collective entity.^44^, ^45^, ^46^ From this point of view, it appears more relevant to emphasize the diminished effectiveness of TNFi in women when compared with bDMARDs of other targets in a specific, well-defined group of patients.

According to current PsA guidelines, preference for IL-23i and IL-17i over TNFi should only be given in patients with severe skin psoriasis.^3^, ^4^, ^5^ In view of usually earlier skin than joint manifestation in PsA,^47^ dermatologists often see patients with PsA earlier than rheumatologists. On the basis of current evidence for a better efficacy on cutaneous psoriasis, dermatologists may already prefer Th17i over TNFi for skin disease. However, based on the large experience with TNFi and their lower costs after patent expiry, TNFi will in the coming years probably remain the first choice of bDMARD therapy worldwide in many cases of PsA. If this assumption holds true, the substantially lower drug retention of an alternative TNFi compared with Th17i in women with PsA in our real-life setting suggests that the patient’s sex should be considered in the second bDMARD line in PsA management.

## Conclusion

We herein report in a comparative real-life effectiveness study in patients with TNFi-experienced PsA that switching to Th17i may provide better effectiveness than cycling to another TNFi in women with PsA. However, when only looking into the patients with follow-up data, TNFi appear to provide better response rates. Thus, after discontinuation of at least one TNFi, both alternative TNFi and Th17i remain viable options. The magnitude of difference in treatment effectiveness in women appears clinically relevant and supports the need for ongoing research efforts on the effect of sex on treatment choices and outcomes, the latter preferentially in large, strategic, sex-stratified, randomized controlled trials for PsA and other spondyloarthritides.

## Potential Competing Interests

The authors report no competing interests.

## Ethics Statement

This study was approved by the cantonal ethics committee in Bern (KEK ID 2019-00968), and all participants provided written informed consent.
