# Supplementary material for: Longer Drug Retention of Interleukin-12/23 or Interleukin-17 Inhibitors Compared With TNF Inhibitors in Female Patients With TNF Inhibitor-Experienced Psoriatic Arthritis
Source: Mayo Clin Proc Innov Qual Outcomes. 2025 May 12;9(3):100622. doi: 10.1016/j.mayocpiqo.2025.100622 (PMC12136923; doi:10.1016/j.mayocpiqo.2025.100622)

Supplemental Material

Supplemental Table 1. DMARD co-medication and treatment history. Frequencies (in parentheses) are with respect to the total TNFi (n=503) or the total Th17i (n=341) TCs.

Drug, DMARD co-medication, history	Absolute numbers	TNFi (n=503)	Th17i (n-341)	All	p-value
**Adalimumab**	n (%)	129 (25.7)			
**Certolizumab Pegol**	n (%)	76 (15.1)			
**Etanercept**	n (%)	105 (20.9)			
**Golimumab**	n (%)	136 (27.0)			
**Infliximab**	n (%)	57 (11.3)			
**Ixekizumab**	n (%)		30 (8.8)		
**Secukinumab**	n (%)		188 (55.1)		
**Ustekinumab**	n (%)		123 (36.1)		
**csDMARD co-therapy**	n (%)	236 (46.9)	128 (37.5)	364 (43.1)	0.007
**bDMARD line**	2nd	230 (45.7)	87 (25.5)	317 (37.6)	
	>= 3rd	273 (54.3)	254 (74.5)	527 (62.4)	<.001
**TNFi history**	n=1	244 (48.5)	106 (31.1)	350 (41.5)	
	n>= 2	259 (51.5)	235 (68.9)	494 (58.5)	<.001
**Th17i history**	yes	57 (11.3)	82 (24)	114 (13.5)	0.0005
**csDMARD history**					
**0**	n (%)	69 (13.7)	39 (11.4)	108 (12.8)	
**1**	n (%)	205 (40.8)	121 (35.5)	326 (38.6)	
**≥ 2**	n (%)	229 (45.5)	181 (53.1)	410 (48.6)	0.09
**bDMARD history**					
**n=1**	n (%)	252 (50.1)	100 (29.3)	352 (41.7)	
**n=2**	n (%)	136 (27.0)	106 (31.1)	242 (28.7)	

**n=3**	n (%)	76 (15.1)	75 (22.0)	151 (17.9)	
**n=4**	n (%)	28 (5.6)	39 (11.4)	67 (7.9)	
**n=5**	n (%)	7 (1.4)	17 (5.0)	24 (2.8)	
**n=6**	n (%)	4 (0.8)	4 (1.2)	8 (0.9)	0.0005
**Ever unapproved bDMARD**	yes	24 (4.8)	28 (8.2)	52 (6.2)	0.06
**Ever tsDMARD**	yes	42 (8.3)	83 (24.3)	125 (14.8)	<0.001
**Reason for stopping the previous bDMARD**					0.0005
**AE**	n (%)	79 (15.7)	45 (13.3)	124 (14.7)	
**Ineffectiveness**	n (%)	259 (51.6)	242 (71.4)	501 (59.6)	
**Other**	n (%)	120 (23.9)	47 (13.9)	167 (19.9)	
**Remission**	n (%)	44 (8.8)	5 (1.5)	49 (5.8)	

Abbreviations: AE: adverse event, b/cs/tsDMARD: biological/conventional synthetic/targeted synthetic Disease-Modifying Anti-Rheumatic drug, Th17i: inhibitors of the Th17 cell generation or function, TNFi: Tumor Necrosis Factor inhibitors. *Limited to reports in SCQM, but csDMARD use before registration is most likely.

Supplemental Table 7 Mean difference and 95% CI of change in continuous secondary outcome parameters from baseline to follow-up, three to 12 months after treatment start with TNFi (n=170) or Th17i (n=122). Data are from cases with complete data for the respective item at treatment start and follow-up while still being on the same bDMARD. The number of TCs with available baseline and follow-up data is indicated by “n”.

	**TNFi**		**Th17i**		**p-value**
	n	Mean (95% CI)	n	Mean (95% CI)	
**CRP (mg/l)**	132	-4.61 (-3.08, -6.13)	94	-4.01 (-2.08, -5.94)	0.81
**DAS28-CRP**	119	-0.68 (-0.57, -0.79)	86	-0.39 (-0.28, -0.51)	0.08
**DAPSA**	56	-8.25 (-6.41, -10.08)	32	-3.47 (-1.42, -5.52)	0.09
**cDAPSA**	70	-7.5 (-5.96, -9.04)	41	-2.95 (-1.28, -4.63)	0.05
**Skin global**[29]	142	-0.52 (-0.4, -0.64)	109	-0.87 (-0.69, -1.05)	0.10
**MASES**	113	-0.54 (-0.33, -0.75)	102	-0.42 (-0.22, -0.62)	0.69
**EQ-5D**	86	5.49 (-7.69, -3.28)	60	2.19 (-4.1, -0.28)	0.26
**HAQ-DI**	86	-0.12 (-0.08, -0.16)	61	-0.09 (-0.04, -0.14)	0.67
**DLQI**	84	-0.76 (-0.23, -1.3)	59	-0.98 (-0.22, -1.74)	0.81

Abbreviations: CI: confidence interval, Th17i: inhibitors of the Th17 cell generation or function, TNFi: Tumor Necrosis Factor inhibitors, CRP: C-reactive protein , DAS28-CRP: Disease Activity Score based on 28 joints and CRP[[illegible]7], (c)DAPSA: (clinical) Disease Activity in PSoriatic Arthritis[26], DLQI: Dermatology Life Quality Index[33], EQ-5D: European Quality of life questionnaire

in five Dimensions HAQ-DI: Health Assessment Questionnaire of Disability Index IQR: interquartile range, MASES: Maastricht Ankylosing Spondylitis Enthesitis Score (with inclusion of the proximal plantar fascia insertions)

Supplemental Table [illegible] Disease activity and health-related quality of life measures three to 12 months after start of an alternative TNFi or Th17i in absolute values.

	**TNFi**		**Th17i**		**p-value**
	n	median (IQR)	n	median (IQR)	
**CRP (mg/l)**	147	4 (1, 5)	106	3.5 (1, 7)	0.89
**DAS28-CRP**	140	2.2 (1.9, 3.3)	99	2.3 (1.8, 3.2)	0.30
**DAPSA**	81	12.1 (4.5, 18.5)	49	10.7 (5.3, 23.2)	0.09
**cDAPSA**	93	12 (4, 18)	53	10 (4, 21)	0.15
**MASES**	146	0 (0, 2)	109	0 (0, 2)	0.84
**Skin global**	155	1 (0, 2)	111	1 (0, 2)	0.20
**EQ-5D**	106	76.6 (66.4, 77.9)	75	69.0 (62.4, 77.9)	0.26
**HAQ-DI**	107	0.50 (0.12, 1.00)	77	0.62 (0.25, 1.12)	0.24
**DLQI**	105	1 (0, 4)	75	1 (0, 5)	0.41

Abbreviations: Th17i: inhibitors of the Th17 cell generation or function, TNFi: Tumor Necrosis Factor inhibitor, CRP: C-reactive protein, DAS28-CRP: Disease Activity Score based on 28 joints and CRP, (c)DAPSA: (clinical) Disease Activity in PSoriatic Arthritis, DLQI: Dermatology Life Quality Index, EQ-5D: European Quality of life questionnaire in five Dimensions, HAQ-DI: Health Assessment Questionnaire of Disability Index, MASES: Maastricht Ankylosing Spondylitis Enthesitis Score (with inclusion of the proximal plantar fascia insertions).

Supplemental Table 4. Distribution of weighted and other variables for IPW.

Variable		TNFi	Th17i	p-value
**Weighted variables**				
**Swollen out of 68 joints**	median (IQR)	1.00 (0.00, 4.00)	1.00 (0.00, 4.00)	0.83
**Painful out of 68 joints**	median (IQR)	3.00 (0.00, 8.00)	4.00 (1.00, 10.00)	0.17
**Active skin disease**	(%)			1.000
**none**		72.3 (23.4)	68.9 (22.9)	
**almost none**		73.1 (23.7)	72.1 (24.0)	
**mild**		68.9 (22.3)	68.0 (22.7)	
**mild/moderate**		31.1 (10.1)	30.3 (10.1)	
**moderate**		34.3 (11.1)	31.5 (10.5)	
**moderate/severe**		23.9 (7.7)	23.7 (7.9)	
**severe**		5.3 (1.7)	5.9 (2.0)	
**MASES**	median (IQR)	0.00 (0.00, 3.00)	1.00 (0.00, 2.00)	0.4
**History of IBD**	(%)	304.5 (98.6)	296.0 (98.6)	0.99
**Uveitis in last 12 months (%)**		307.9 (99.7)	300.3 (100.0)	0.33
**bDMARDs line >= 3rd (%)**		189.0 (61.2)	182.9 (60.9)	0.96
**Previous drug type = TNFi**	(%)	262.6 (85.0)	258.0 (85.9)	0.85
**Other variables**				
**Female sex**	(%)	210.6 (68.2)	184.0 (61.3)	0.24
**Age [years]**	median (IQR)	50.18 (38.00, 61.00)	53.00 (45.05, 59.00)	0.15
**Smoking status**	(%)			0.64
**Former smoker**		72.2 (41.9)	41.5 (34.2)	
**Current smoker**		40.1 (23.3)	28.9 (23.8)	

**Physical activity groups**	(%)			0.77
**No physical activity**		44.4 (26.0)	42.2 (34.8)	
**Less than 1 hour per week**		35.2 (20.6)	23.6 (19.5)	
**1 to 2 hours per week**		63.6 (37.3)	39.0 (32.1)	
**More than 2 hours per week**		27.4 (16.0)	16.6 (13.7)	
**Positive MDA status**	(%)	224.3 (82.4)	238.8 (89.6)	0.12
**HLA-B27**	(%)	148.3 (83.6)	162.5 (86.3)	0.63
**CASPAR positive**	(%)	99.5 (32.2)	60.6 (20.2)	0.04
**Dactylitis ever**	(%)	175.5 (56.8)	144.8 (48.2)	0.16
**Nail manifestations ever**	(%)	249.2 (80.7)	193.8 (64.5)	<.01
**Enthesitis ever**	(%)	53.4 (17.3)	54.6 (18.2)	0.85
**Active dactylitis**	(%)	284.0 (91.9)	269.3 (89.7)	0.51
**Active enthesitis**	(%)	159.3 (51.6)	129.6 (43.2)	0.17
**csDMARD co-therapy**	(%)	177.5 (57.5)	177.9 (59.2)	0.77
**Cardiovascular event ever**	(%)	205.6 (66.6)	174.2 (58.0)	0.15
**Fibromyalgia**	(%)	303.4 (98.2)	283.3 (94.3)	0.08
**BMI [kg/m^2]**	mean (SD)	28.51 (5.16)	27.89 (6.01)	0.12
**Disease duration [years]**	median (IQR)	10.00 (5.00, 16.00)	11.00 (5.77, 17.00)	0.39
**HAQ-DI**	mean (SD)	0.73 (0.63)	0.81 (0.60)	0.25
**DLQI**	median (IQR)	2.00 (0.00, 7.57)	1.00 (0.00, 8.00)	0.57
**DAS28-CRP**	median (IQR)	2.70 (1.90, 3.70)	3.10 (2.30, 3.78)	0.08
**EQ-5D**	mean (SD)	64.71 (21.71)	65.10 (21.76)	0.73
**SF-12 MCS**	mean (SD)	43.87 (11.54)	43.57 (11.48)	0.90

**SF-12 PCS**	mean (SD)	40.56 (10.50)	39.41 (9.33)	0.45

Abbreviations: IPW: Inverse Propensity Score-weighted, TNFi: Tumor Necrosis Factor inhibitors, Th17i: inhibitors of the Th17 cell generation or function, IQR: interquartile range, MASES: Maastricht Ankylosing Spondylitis Enthesitis Score (with inclusion of the proximal plantar fascia insertions, IBD: Inflammatory Bowel Disease, bDMARDs: biological Disease-Modifying Anti-Rheumatic Drugs, MDA: Minimal Disease Activity, HLA: Human Leukocyte Antigen, CASPAR: Classification Criteria for Psoriatic Arthritis, csDMARD: conventional synthetic Disease-Modifying Anti-Rheumatic Drugs, BMI: Body Mass Index, HAQ-DI: Health Assessment Questionnaire of Disability Index, DLQI: Dermatology Life Quality Index, DAS28-CRP: Disease Activity Score based on 28 joints and C-reactive protein, EQ-5D: European Quality of life questionnaire in five Dimensions, SF-12: Short Form questionnaire with 12 items, MCS: Mental Component Score, PCS: Physical Component Score

Supplemental Figure 1. Competing risks analysis and cumulative incidence for the different treatment discontinuation reasons. **A.** In TNFi. **B.** In Th17i.

A. Reasons for TNFi discontinuation.

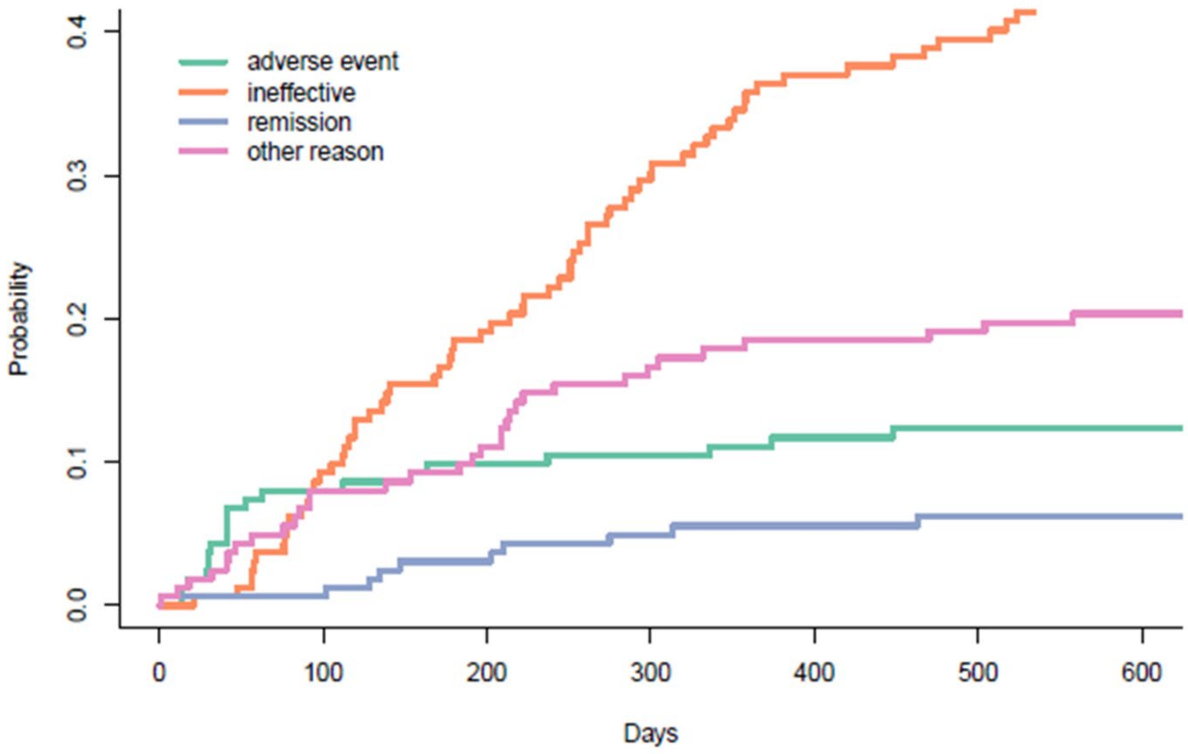

B. Reasons for Th17i discontinuation.

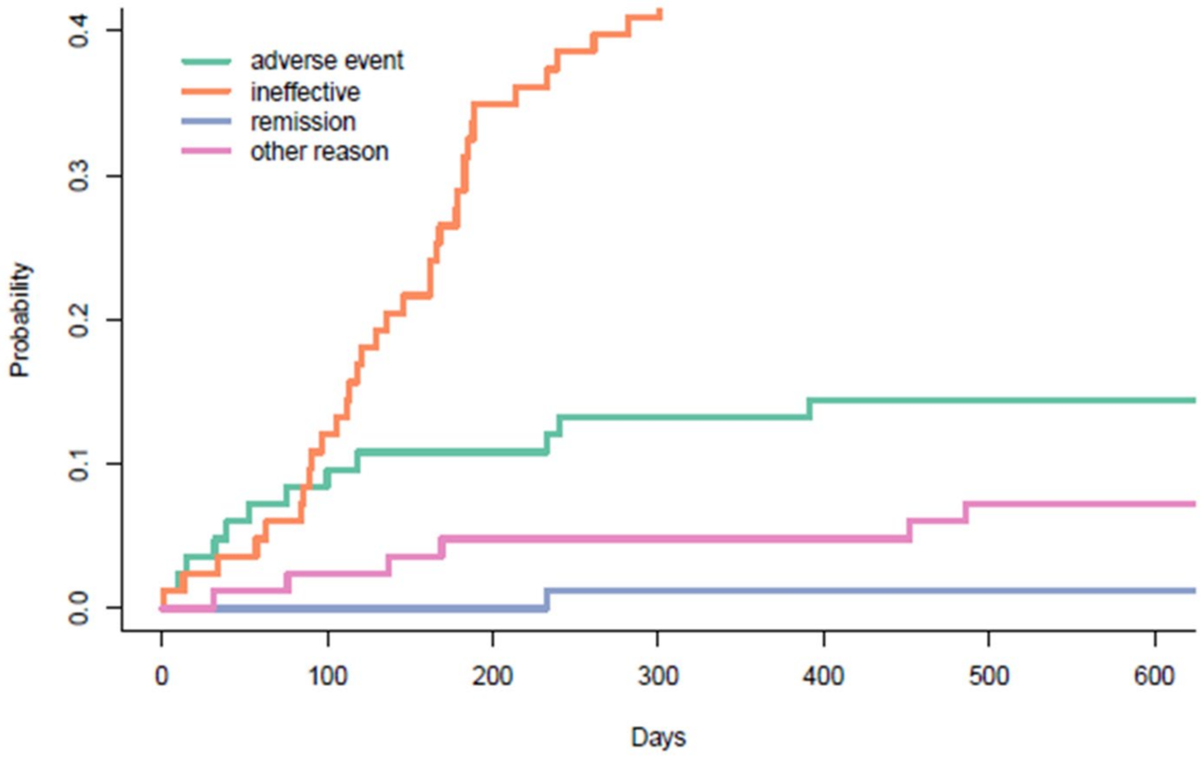

Supplement: Supplemental Tables [file mmc1.pdf]
